# The Impact of Hypothyroidism on the Quality of Life of Adults in Riyadh, Saudi Arabia

**DOI:** 10.7759/cureus.37636

**Published:** 2023-04-16

**Authors:** May I AlAwaji, Rana H Alhamwy

**Affiliations:** 1 Family Medicine, King Fahad Medical City, Riyadh, SAU

**Keywords:** subclinical hypothyroidism, hypothyroidism, thyroid-stimulating hormone (tsh), health related-quality of life, whoqol-bref, quality-of-life, thyroid gland

## Abstract

Background and objective

Hypothyroidism is extremely common and associated with far-reaching health consequences. The negative effects of hypothyroidism on patients' quality of life (QoL) are well-documented. It is said to be common in the countries of the Arabian Gulf, although it is often misdiagnosed and treated in an inconsistent manner. Hence, understanding how an illness like this affects a patient's life might help us enhance their QoL and get us closer to the healthcare transformation goals of Saudi Arabia's Vision 2030.

Methodology

This cross-sectional study was conducted in Riyadh, Saudi Arabia, between June 2022 and February 2023. A convenience non-probability sampling method was used. The WHO Quality of Life (WHOQOL)-BREF questionnaire in Arabic was used to compile the data. Data were collected using a standardized form, refined using Google Forms, and then documented in an Excel spreadsheet. The descriptive statistics were shown as means and standard deviations (SD). To assess the numerical data, a t-test was used, while the chi-square test was employed to examine the relationship between the qualitative factors.

Results

A total of 394 adults from the general population with hypothyroidism were surveyed, including 105 men and 289 women. Of them, 151 (38.3%) patients had not sought therapy for their hypothyroidism, while 243 (61.7%) patients had. When asked about the QoL, a significant segment (37.6%) of patients reported that it was high, and 29.7% reported being totally satisfied with their health. The WHOQOL-BREF domain scores revealed that environmental health had the highest value (24.04 ±4.62), followed by physical health (22.24 ±3.23), and then psychological health (18.08 ±2.82), and the lowest scores were reported for the rate of QoL and satisfaction with health (2.64 ±1.36 and 2.80 ±1.68), respectively. Each domain of the WHOQOL-BREF had its own set of variables that differed from one another in a statistically significant manner (p<0.001).

Conclusions

Based on our findings, we recommend expert physician monitoring and implementing educational programs as well as placing a greater emphasis on patients' QoL in the management of hypothyroidism.

## Introduction

Hypothyroidism is widely prevalent and has far-reaching effects on patient health. It is well established that hypothyroidism is linked to various undesirable health consequences that substantially affect patients' quality of life (QoL) [1]. These patients report that they typically experience higher reductions in physical dimensions, compared to social and emotional dimensions [2]. From an epidemiological perspective [3], it appears that the incidence of (usually moderate) hypothyroidism is on the rise. Hypothyroidism can develop due to a number of reasons, including primary gland failure or inadequate stimulation of the thyroid gland by the hypothalamus or pituitary gland [4]. In addition, it can also result from a deficiency in the production of thyroid hormone by the thyroid gland due to multiple etiologies [5]. Patients with hypothyroidism can have symptoms that impair their health status and QoL [6]. This disorder slows the metabolism, with classic symptoms of low energy, tiredness, change in appearance, weight gain, and cold intolerance; easy fatigability is a common symptom as well [7].

The prevalence of the condition ranges from 0.2 to 5.3% in Europe [8], with rates reaching as high as 17% among elderly people [9]; adult women are disproportionately affected by thyroid problems, with hypothyroidism having a prevalence of almost 10 times higher in women than in males [8]. Hypothyroidism is also reported to be widespread in the Arabian Gulf States; however, it is often misdiagnosed and treated in an inconsistent manner [10]. No population-wide studies of hypothyroidism have been conducted in Saudi Arabia. However, in a cross-sectional study conducted in outpatient clinics of a tertiary care hospital in Riyadh that included 463 patients between December 2018 and December 2019, primary hypothyroidism prevalence was found to be 47.8% [11]. Furthermore, cross-sectional research from Riyadh found a prevalence of subclinical hypothyroidism of 10% among people visiting a primary care clinic [12]. Similarly, in another study conducted in Saudi Arabia, it was estimated that the prevalence of hypothyroidism was 18.7%; the feeling of lethargy and laziness was found in 72.9%, and mood changes in 69.4% of cases [13]. Moreover, subclinical hypothyroidism was shown to be prevalent among a sample of 567 Saudis (15.9%) in a study conducted in the Al Bahah region [14].

QoL is defined as the feeling of well-being regarding multiple aspects of life [15]. WHO has defined it as "an individual’s perception of their position in life in the context of the culture and value system in which they live, and in relation to their goals, expectations, standards, and concerns" [15]. Recent research has shown that people with hypothyroidism typically have a lower health-related QoL compared to the general population [2]. Some individuals with hypothyroidism who are on levothyroxine (L-T4) still express concern about their QoL [16]. It has been found that the negative impact of hypothyroidism on QoL is higher in patients with poorer mental health [17], and depression is one of the most common psychiatric characteristics associated with hypothyroidism in elderly people [18]. Moreover, QoL has been observed to decrease with increasing weight in previous research among hypothyroidism patients who had undergone treatment [16]. In addition, some patients with hypothyroidism experience a goiter-related enlargement of the front neck [19]. Congenital iodine deficiency syndrome and other birth defects can occur if hypothyroidism during pregnancy is not treated [20]. Unfortunately, there are scarce studies investigating QoL in patients with hypothyroidism in Saudi Arabia. A case-control study performed at King Abdulaziz University Hospital reported that patients with hypothyroidism have worse physical health than the healthy controls, which has a negative impact on patients' QoL and contributed to a diminished standard of living [1]. In light of the significance of these findings, more attention must be paid to these health-related issues in order to achieve the goals of Saudi Arabia's Vision 2030.

One of the most important healthcare transformation goals in Saudi Arabia’s Vision 2030 involves improving the well-being and the QoL of Saudi citizens [21], and as family physicians, it is our primary role to help improve our patients' QoL [22]. To our knowledge, very limited research has been conducted in Saudi Arabia to assess the QoL in adult patients with hypothyroidism, despite the fact that there have been a number of reports on the frequency and risk factors of hypothyroidism. Given the dearth of data on the effects of hypothyroidism on QoL, this study sought to address the issue. Knowing the impact of such a disease on a patient’s life can help us improve their QoL and contribute to achieving the healthcare transformation goals of Saudi Arabia’s Vision 2030.

## Materials and methods

This was a cross-sectional observational study conducted in Riyadh, Saudi Arabia from June 2022 to February 2023. The appropriate sample size was determined using the following formula [23]:

n=Z2P(1-P)/e2

Where n is the sample size, Z is the statistic corresponding to the level of confidence, P is the expected prevalence (obtained from identical studies or a pilot study done by the researchers), and e stands for precision (corresponding to effect size). Most researchers provide their findings with a 95% confidence interval (CI), which is the standard level of confidence to aim for. However, some researchers choose a 99% confidence interval if they wish to be more certain. In order to use the formula, the researcher needs to be aware of the assumed P [24]. However, we used a confidence interval of 95% and a margin of error of 5%, and after calculations, the sample size was estimated to be 394. Furthermore, a convenience non-probability sample was used, and a link to the online questionnaire was sent to potential participants in Riyadh, Saudi Arabia.

Inclusion and exclusion criteria

Only Saudi patients aged 18-60 years old and diagnosed with hypothyroidism were included in the present study. Patients aged less than 18 years or more than 60 years and those not known to have hypothyroidism were excluded.

Data collection

Data was collected using the Arabic version of the WHO's QoL (WHOQOL-BREF) questionnaire [25]. The WHOQOL-BREF is a 26-item tool that assesses general health and QOL in four distinct areas: physical health (seven items), mental health (six items), social relationships (two items), and environmental health (eight items). Using a response scale that is mandated as a 5-point ordinal scale, the WHOQOL-BREF items are rated on a scale of 1 to 5 [26].

Ethical approval

Ethical approval was obtained from King Fahad Medical City (KFMC) Institutional Review Board, Riyadh, Saudi Arabia on August 1, 2022 (reference no. 22-330).

Data analysis

All information was recorded on a standardized form, processed further using Google Forms, and then organized in an Excel file. The descriptive data were presented as a mean ±standard deviation (SD). The t-test was used to analyze the quantitative data, and the chi-square test was used to examine the correlation between the qualitative variables. A p-value <0.05 was considered statistically significant. In light of the strong associations found by the univariate analysis, we also employed multivariate analysis by using the statistical software IBM SPSS Statistics version 23 (IBM Corp., Armonk, NY).

## Results

Demographic and clinical characteristics

A total of 394 community-dwelling adults (105 males and 289 females) participated in this study. The mean age of the cohort was 34.74 ±9.31 years (p<0.001); 236 (59.1%) of the respondents were married, while 6.9% (n=27) were divorced (p<0.001). No foreigners were included in the analysis, and all respondents were residents of Riyadh; 243 patients (61.7%) had sought treatment for hypothyroidism, while 151 patients (38.3%) had not (p<0.001), as indicated in Table 1.

**Table 1:** Demographic and clinical characteristics SD: standard deviation

Variables	Category	Values	Chi-square	P-value
Gender, n (%)	Male	105 (26.6)	85.93	0.001
	Female	289 (73.4)		
Age in years	Mean ±SD	34.74 ±9.31	271.82	0.001
Marital status, n (%)	Single	131 (33.2)	166.30	0.001
	Married	236 (59.9)		
	Divorced	27 (6.9)		
Treatment for hypothyroidism, n (%)	Yes	243 (61.7)	21.48	0.001
	No	151 (38.3)		

Rating quality of life

**Figure 1:**
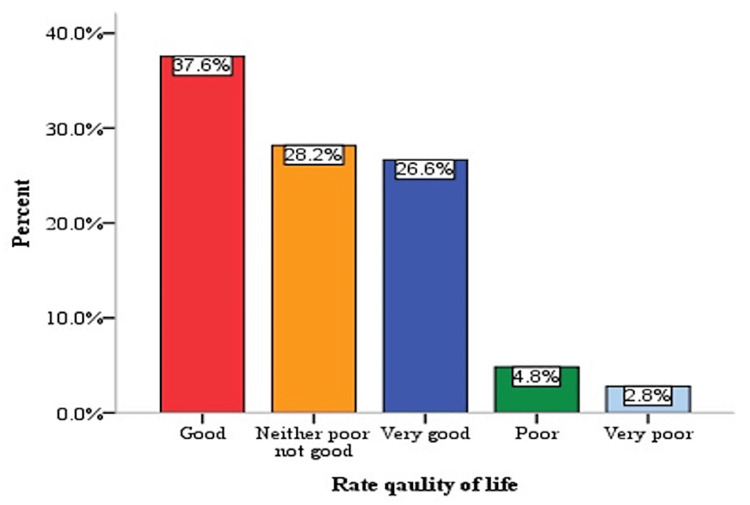
Descriptive analysis to rate the quality of life among study participants

When asked "How would you assess your quality of life?", a high number of participants (37.6%) responded that it was "good", 28.2% responded that it was "neither poor nor good", 26.6% responded that it was "very good", and only 2.8% responded that it was "very poor" while 4.8% reported that their quality of health was "poor" (Figure 1).

Satisfaction with health

**Figure 2:**
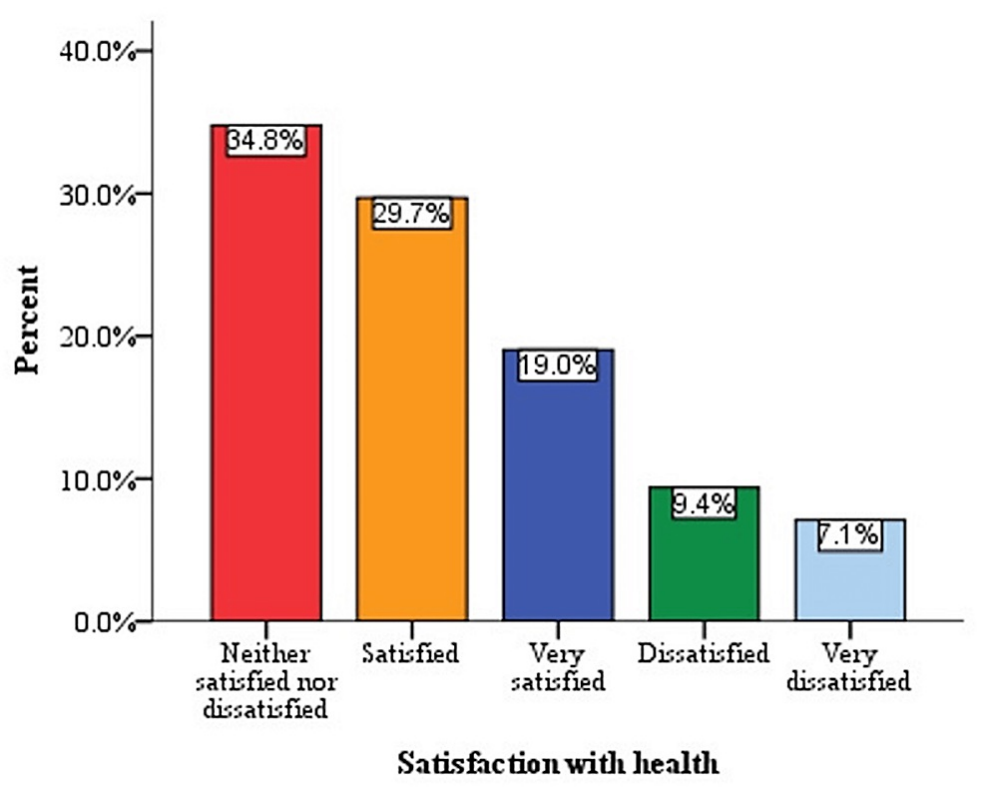
Level of satisfaction with health

There was a wide range of responses to the question related to participant satisfaction with their health, with 19% indicating they were "very satisfied", 29.7% indicating satisfaction, 34.8% indicating they were "neither satisfied nor dissatisfied", 9.4% indicating dissatisfaction, and 7.1% indicating they were "very dissatisfied" (Figure 2).

Quality of life among the participants

The WHOQOL-BREF domain scores are shown in Table 2. The environmental health domain had the highest mean score (24.04 ±4.62) among the WHOQOL-BREF dimensions, followed by the physical health domain (22.24 ±3.23) and finally the psychological health domain (18.08 ±2.82), with the lowest mean scores reported in the rate of QoL and satisfaction with health (2.64 ±1.36 and 2.80 ±1.68), respectively. The majority of participants had a relatively low QoL assessment value and health satisfaction, as indicated by the fact that the median score was lower than the midpoint of the measurement scale of responses in all WHOQOL-BREF domains, as shown in Table 2.

**Table 2:** Overall WHOQOL-BREF domains scores WHOQOL: World Health Organization Quality-of-Life Scale

Domains	Number of items	Mean score ±SD	Median	Minimum	Maximum
Physical health	7	22.24 ±3.23	22.00	13.00	30.00
Psychological health	6	18.08 ±2.82	18.00	10.00	27.00
Social relationships	2	6.46 ±2.36	6.00	2.00	10.00
Environmental health	8	24.04 ±4.62	24.00	13.00	34.00
Rate of quality of life	1	2.64 ±1.36	2.00	1.00	5.00
Satisfaction with health	1	2.80 ±1.68	2.00	1.00	5.00

WHOQOL-BREF scores of patients with hypothyroidism

As shown in Table 3, in the domain of physical health, the majority 139 (35.3%) reported having a little physical pain, followed by 126 (32%) reporting a moderate amount of physical pain, while 1.3% of participants reported an extreme amount of physical pain (χ^2^=156.24, p=0.001). Meanwhile, 35.3% stated that they had received a little medical treatment for hypothyroidism, followed by 24.9% who had received no treatment at all, while 3.8% said they had received an extreme amount of medical treatment (χ^2^=116.15, p=0.001). In terms of energy, 40.6% of participants had moderate energy while 2.3% did not (χ^2^=204.147, p=0.001). Regarding physical health in terms of the capacity to work, the majority (42.6%) were moderately satisfied with their physical health, while 10.9% (43) were very satisfied (χ^2^=170.62, p=0.001).

**Table 3:** Analysis of WHOQOL-BREF domains in patients with hypothyroidism SD: standard deviation; WHOQOL: World Health Organization Quality-of-Life Scale

Variable	Response, n (%)					Mean	SD	P-value
Physical health	An extreme amount	Very much	A moderate amount	A little	Not at all			
Physical pain	5 (1.3)	47 (11.9)	126 (32.0)	139 (35.3)	77 (19.5)	3.60	0.97	(χ^2^=156.24, p=0.001)
Medical treatment	15 (3.8)	93 (23.6)	49 (12.24)	139 (35.3)	98 (24.9)	3.54	1.20	(χ^2^=116.15, p=0.001)
	Not at all	A little	Moderately	Mostly	Completely			
Enough energy	9 (2.3)	130 (33.0)	160 (40.6)	52 (13.2)	43 (10.9)	2.97	0.99	(χ^2^=204.147, p=0.001)
	Very good	Good	Moderately	A little	Very poor			
Get around	158 (40.1)	131 (33.2)	86 (21.8)	16 (4.1)	3 (0.8)	1.92	0.91	(χ^2^=237.80, p=0.001)
	Very satisfied	Very dissatisfied	Moderately satisfied	Dissatisfied	Satisfied			
Sleep satisfaction	28 (7.1)	12 (3.0)	185 (47.0)	64 (16.2)	105 (26.6)	3.52	1.13	(χ^2^=243.99, p=0.001)
Daily living activities	39 (9.9)	20 (5.1)	203 (51.5)	51 (12.9)	81 (20.6)	3.29	1.15	(χ^2^=269.60, p=0.001)
Capacity to work	43 (10.9)	17 (4.3)	169 (42.9)	73 (18.5)	92 (23.4)	3.39	1.20	(χ^2^=170.62, p=0.001)
Psychological health	An extreme amount	Very much	A moderate amount	A little	Not at all			
Life enjoyment	47 (11.9)	96 (24.4)	165 (41.9)	80 (20.3)	6 (1.5)	2.75	0.96	(χ^2^=178.16, p=0.001)
Meaningful life	84 (21.3)	122 (31.0)	124 (31.5)	45 (11.4)	19 (4.8)	2.47	1.094	(χ^2^=109.83, p=0.001)
Able to concentrate	39 (9.9)	72 (18.3)	162 (41.1)	108 (27.4)	13 (3.3)	2.96	0.99	(χ^2^=174.29, p=0.001)
	Not at all	A little	Moderately	Mostly	Completely			
Bodily appearance	0	0	144 (36.5)	142 (36.0)	108 (27.4)	3.91	0.79	(χ^2^=6.23, p=0.044)
	Very satisfied	Very dissatisfied	Moderately satisfied	Dissatisfied	Satisfied			
Satisfaction with yourself	68 (17.3)	20 (5.1)	177 (44.9)	48 (12.2)	81 (20.6)	3.14	1.29	(χ^2^=179.83, p=0.001)
	Always	Very often	Quite often	Seldom	Never			
Negative feeling	38 (9.6)	108 (27.4)	137 (34.8)	94 (23.9)	17 (4.3)	2.86	1.03	(χ^2^=126.33, p=0.001)
Social relationships	Very satisfied	Very dissatisfied	Moderately satisfied	Dissatisfied	Satisfied			
Personal relationships	68 (17.3)	50 (12.7)	104 (26.4)	66 (16.8)	106 (26.9)	3.23	1.42	(χ^2^=31.53, p=0.001)
Support from friends	58 (14.7)	35 (8.9)	158 (40.1)	44 (11.2)	99 (25.1)	3.23	1.32	(χ^2^=129.98, p=0.001)
Environmental health	An extreme amount	Very much	A moderate amount	A little	Not at all			
Feeling of safety	83 (21.1)	100 (25.4)	95 (24.1)	102 (25.9)	14 (3.6)	2.65	1.18	(χ^2^=69.38, p=0.001)
Physical environment	61 (15.5)	91 (23.1)	138 (35.0)	80 (20.3)	24 (6.1)	2.78	1.11	(χ^2^=88.51, p=0.001)
	Not at all	A little	Moderately	Mostly	Completely			
Money for needs	22 (5.6)	65 (16.5)	167 (42.4)	93 (23.6)	47 (11.9)	3.19	1.03	(χ^2^=157.72, p=0.001)
Information in daily life	11 (2.8)	95 (24.1)	137 (34.8)	102 (25.9)	49 (12.4)	3.21	1.03	(χ^2^=122.75, p=0.001)
Leisure activities	40 (10.2)	90 (22.8)	128 (32.5)	106 (26.9)	30 (7.6)	2.98	1.10	(χ^2^=91.02, p=0.001)
	Very satisfied	Very dissatisfied	Moderately satisfied	Dissatisfied	Satisfied			
Satisfaction with living place	115 (29.2)	34 (8.6)	86 (21.8)	38 (9.6)	121 (30.7)	3.04	1.61	(χ^2^=86.48, p=0.001)
Satisfaction with health services	95 (24.1)	32 (8.1)	124 (31.5)	45 (11.4)	98 (24.9)	3.05	1.47	(χ^2^=76.28, p=0.001)
Satisfaction with transport	95 (24.1)	26 (6.6)	126 (32.0)	33 (8.4)	114 (28.9)	3.11	1.50	(χ^2^=109.33, p=0.001)

There were six variables in the domain of psychological health. With regard to the variable of life enjoyment, 41.9% of participants stated they moderately enjoyed their life and six (1.5%) said they did not enjoy their life (χ^2^=178.16, p=0.001). In terms of having a meaningful life and being able to concentrate, a large number of participants responded that they had these variables in moderate amounts (31.5% and 41.1%, respectively) (p=0.001). Meanwhile, 36.5% stated they had a moderate bodily appearance (p=0.04). There was a significant (p=0.001) difference in terms of participants' satisfaction with life; 34.8% of participants said they quite often had negative feelings (χ^2^=126.33, p=0.001).

There were two variables in the domain of social health: personal relationships and support from friends; 26.9% of the participants were satisfied with their personal relationships while 40.1% were moderately satisfied with support from friends (p=0.001).

In the domain of environmental health, there were eight variables; 25.9% of participants expressed having a little feeling of safety, while 35% stated that the physical environment had a moderate amount of impact on environmental health (p=0.001). With regard to the variables of money for needs, information in daily life, and leisure activity, most of the participants stated that they had these variables in moderate amounts (42.2%, 34.8%, and 32.5%, respectively) (p=0.001). Furthermore, 30.7% were satisfied with their living place, while 31.5% and 32% were moderately satisfied with the health services and transport, respectively (p=0.001).

## Discussion

Hypothyroidism is reported to be widespread in the Arabian Gulf States; however, it is frequently misdiagnosed, and its treatment is inconsistently administered [10]. In light of this, the present study aimed to assess the impact of this condition on patient life.

In the present study, all participants had hypothyroidism, and 61.7% had received some treatment for the condition, while 38.3% had received no treatment. The fact that some patients had not received any treatment could be attributed to various factors, such as negligence or ignorance, or some patients manifesting mildly increased thyroid stimulating hormone (TSH) levels and no other symptoms and hence not requiring any treatment. TSH levels must be quite high (above 10 mU/L) before many doctors consider medication, and there are other predictors as well, such as the overall risk of cardiovascular disease, that may play a role in the decision [27]. Careful monitoring of the patient's reaction to thyroid hormone replacement treatment is crucial to the management of hypothyroidism. The elderly and patients at risk for cardiac issues are the two groups who benefit most from starting hormone replacement therapy at a modest dosage [28].

Untreated hypothyroidism was associated with a decrease in QoL, most noticeably fatigue, although QoL was enhanced post-treatment. Several factors contribute to this, such as the individual's need to take medication on a regular basis and the presence of a relevant medical diagnosis and overtreatment [3]. In the current study, when participants were asked about QoL, 37.6% reported having a good QoL, while only 2.8% had very poor and 4.8% had poor QoL. A high percentage of participants had good QoL because most of the participants had received some treatment, which enhanced their QoL. These findings are in line with those of other studies, which concluded that the median ThyPRO-39 QoL score after 12 months of L-T4 therapy was substantially higher [29]. In a meta-analysis of 21 randomized clinical trials involving 2192 patients with subclinical hypothyroidism, it was found that some treatments may not give the desired results and improve the QoL, as thyroid hormone therapy was not associated with substantial improvements in overall QoL (standardized mean difference: 0.11) or improvement in thyroid-related symptoms [30]. In a survey that included 406 people (380 women and 26 men), the mHealth app was found to have a significant impact on QoL. The vast majority of participants (95.8%) felt that using the app was beneficial, with 68% citing an improvement in their QoL and 70% citing a favorable influence on their health. Less severe manifestations of symptoms were reported by those who found the app helpful. Many individuals cited the app's health information as a major factor in their improved ability to manage their health [31].

Meanwhile, 29.7% of the participants were satisfied with their health, which may be ascribed to the timely treatment they had received, and they were enjoying good QoL, while 9.4% and 7.1% indicated they were dissatisfied and very dissatisfied with their health, respectively. It is unsurprising that many patients feel unhappy because they report different sets of symptoms, some of which can be definitively linked to hypothyroidism and others that cannot [32]. Patients with hypothyroidism and normal TSH levels were more likely to report memory problems, trouble finding the right words, fatigue, weight gain, sore muscles and joints, foggy thinking, inability to concentrate, and frequent accidents when walking or using objects, according to a community-based survey [33].

The WHO has developed a shortened version of the WHOQOL-100 questionnaire called WHOQOL-BREF. The physical, psychological, social, and environmental dimensions of QoL are all addressed by this instrument [34]. The WHOQOL-BREF is considered the most effective tool to measure QoL because it does not place undue stress on the respondent [34]. In the present study, a high number of participants had relatively low QoL assessment levels, when asked to rate QoL, and health satisfaction, as indicated by the fact that the median score was lower than the midpoint of the measurement scale of responses (Table 2). In contrast, in one study, patient QoL was not significantly different from that of healthy individuals in the test and control groups (p>0.001). Yet, there was a statistically significant difference between the mental health levels of patients (59.70) compared to those of normal people (48.68) across all measures (p<0.001) [35]. Furthermore, patients' mean WHOQOL-26 scores were 3.51 ±0.43 for males and 3.59 ±0.42 for women; these values did not differ significantly from those of healthy people (3.32 ±0.42 for men and 3.35 ±0.49 for women). On the WHOQOL-26, no statistically significant differences were found between sick and healthy people. In the present study, the environmental health domain had a mean score of 24.04 ±4.62 followed by the physical health domain (22.24 ±3.23) and the psychological health domain (18.08 ±2.82) among the WHOQOL-BREF dimensions. However, according to the results of another study that looked at the correlation between hypothyroidism and QoL in relation to body mass index (BMI), patients with hypothyroidism reported lower QoL and more difficulties with physical functioning [16]. A study conducted in India in 2018 showed that those with hypothyroidism had lower QoL scores across the board, notably in physical health [36]. Similarly, a study by Martino et al. in 2021 observed that there is no connection between QoL indices and anxiety symptoms (psychological domain) [37].

However, the question may arise as to why the participants had low QoL scores for the rate of QoL and satisfaction with health. This could be mainly related to the fact that the majority of the study participants were on treatment, and all treatments had significant effects, apart from the other associated comorbidities the patients may have; however, Saudi people already have good QoL in general [38]. Similarly, according to Winther and Cramon [17], people with hypothyroidism had worse overall QoL scores, especially in the area of mental health. As an additional piece of evidence, the findings of Bathla et al. showed that patients with hypothyroidism are more likely to experience depressive and anxious symptoms than healthy controls [39]. In addition, Han et al. [40] found that people with hypothyroidism in the Korean population actually had a higher HRQoL than euthyroid people.

Although this study has its significance as very limited research has been conducted in this field, it has several limitations. Primarily, there was no comparison or control group, and including one would have led to a better understanding of the reasons for low or high QoL and satisfaction with health. Secondly, there were not enough data regarding the treatment or other comorbidities, which has an impact and influence on the QoL. Future research should incorporate additional parameters such as comorbidities. In addition, blood biochemical analysis for TSH and treatments received by the participants should also be considered for more insightful study outcomes.

Recommendations

Education programs should be initiated for patients with hypothyroidism to combat this health risk in a better way. Biochemical screening programs should be conducted to identify non-symptomatic hypothyroidism. Use of mobile health (mHealth) apps should be encouraged, which can be helpful in enhancing QoL. Efforts should be made to implement the most appropriate treatment methods as certain hormonal therapy can sometimes cause complications in case of comorbidity of cardiovascular diseases. Before starting treatment, alternatives such as lifestyle changes should be considered. Weight is a very important risk factor for hypothyroidism, and hence proper physical exercise should be encouraged to prevent weight gain. Guidelines in the context of local needs for physicians as well as patients should be developed. Steps to increase the level of comfort between the healthcare provider and the patient and keep the therapeutic connection healthy should be implemented. Instituting counseling programs regarding treatment, especially for pregnant women, should be considered.

## Conclusions

It is well known that hypothyroidism is a significant health risk, and its impact on people's QoL is substantial. The WHO defines QoL as an individual's appraisal of their circumstances in light of their own values and those of the society in which they find themselves. Based on our findings, patients with hypothyroidism had higher scores in terms of environmental, physical, and psychological health but showed a decrease in the rate of QoL and health satisfaction. These results highlight the need for increased focus on patients' QoL when managing hypothyroidism, as well as the implementation of expert physician monitoring and educational initiatives.

## References

[1] Ghamri R, Babaker R, Ezzat S (2022). Assessment of quality of life among patients with primary hypothyroidism: a case-control study. Cureus.

[2] Shivaprasad C, Rakesh B, Anish K, Annie P, Amit G, Dwarakanath CS (2018). Impairment of health-related quality of life among Indian patients with hypothyroidism. Indian J Endocrinol Metab.

[3] Hegedüs L, Bianco AC, Jonklaas J, Pearce SH, Weetman AP, Perros P (2022). Primary hypothyroidism and quality of life. Nat Rev Endocrinol.

[4] Gaitonde DY, Rowley KD, Sweeney LB (2012). Hypothyroidism: an update. Am Fam Physician.

[5] Almandoz JP, Gharib H (2012). Hypothyroidism: etiology, diagnosis, and management. Med Clin North Am.

[6] Jaeschke R, Guyatt G, Cook D, Harper S, Gerstein HC (1994). Spectrum of quality of life impairment in hypothyroidism. Qual Life Res.

[7] McMillan C, Bradley C, Razvi S, Weaver J (2008). Evaluation of new measures of the impact of hypothyroidism on quality of life and symptoms: the ThyDQoL and ThySRQ. Value Health.

[8] Chaker L, Bianco AC, Jonklaas J, Peeters RP (2017). Hypothyroidism. Lancet.

[9] Taylor PN, Albrecht D, Scholz A, Gutierrez-Buey G, Lazarus JH, Dayan CM, Okosieme OE (2018). Global epidemiology of hyperthyroidism and hypothyroidism. Nat Rev Endocrinol.

[10] Alzahrani AS, Al Mourad M, Hafez K (2020). Diagnosis and management of hypothyroidism in Gulf Cooperation Council (GCC) countries. Adv Ther.

[11] Alrudian N, Parameaswari P, Alqahtani R (2022). Quality of life in outpatient primary hypothyroidism in Riyadh. Pak J Med Health Sci.

[12] Al Eidan E, Ur Rahman S, Al Qahtani S, Al Farhan AI, Abdulmajeed I (2018). Prevalence of subclinical hypothyroidism in adults visiting primary health-care setting in Riyadh. J Community Hosp Intern Med Perspect.

[13] Alqahtiani NM, Alramadhan ZT, bin Obaid MR (2020). Hypothyroidism in Saudi Arabia; prevalence, risk factors, and its relation with diabetes mellitus. Arch Pharm Pract.

[14] Fureeh AA, Al-Ghamdi AH, Alhuussaini JT (2019). Prevalence and risk factors of subclinical thyroid disorders in Al-Baha Region, Saudi Arabia. Epidemiology.

[15] (1995). The World Health Organization Quality of Life assessment (WHOQOL): position paper from the World Health Organization. Soc Sci Med.

[16] Kelderman-Bolk N, Visser TJ, Tijssen JP, Berghout A (2015). Quality of life in patients with primary hypothyroidism related to BMI. Eur J Endocrinol.

[17] Winther KH, Cramon P, Watt T (2016). Disease-specific as well as generic quality of life is widely impacted in autoimmune hypothyroidism and improves during the first six months of levothyroxine therapy. PLoS One.

[18] Chueire VB, Romaldini JH, Ward LS (2007). Subclinical hypothyroidism increases the risk for depression in the elderly. Arch Gerontol Geriatr.

[19] So M, MacIsaac RJ, Grossmann M (2012). Hypothyroidism: investigation and management. Aust Fam Physician.

[20] Braverman LE, Cooper D (2012). Werner & Ingbar's The Thyroid: A Fundamental and Clinical Text. https://books.google.com.sa/books?hl=en&lr=&id=DaNIXqNLmXsC&oi=fnd&pg=PR3&dq=Werner+%26+Ingbar%27s+the+thyroid:+a+fundamental+and+clinical+text&ots=6s1Jb3fOkx&sig=DOFmGYZ4EyRn1QQYOAApyxkiNfM&redir_esc=y#v=onepage&q=Werner%20%26%20Ingbar's%20the%20thyroid%3A%20a%20fundamental%20and%20clinical%20text&f=false.

[21] (2023). Ministry Of Health Saudi Arabia: Healthcare Transformation Strategy. https://www.moh.gov.sa/en/Ministry/vro/Pages/Health-Transformation-Strategy.aspx.

[22] Phillips WR, Haynes DG (2001). The domain of family practice: scope, role, and function. Fam Med.

[23] Daniel WW, Cross CL (1999). Biostatistics: a Foundation for Analysis in the Health Sciences. https://books.google.com.sa/books?hl=en&lr=&id=PON1DwAAQBAJ&oi=fnd&pg=PA3&dq=Biostatistics:+a+foundation+for+analysis+in+the+health+sciences&ots=a72r8RqqNw&sig=aTcLjCPCJ6LDAnDmqStnck7cHh4&redir_esc=y#v=onepage&q=Biostatistics%3A%20a%20foundation%20for%20analysis%20in%20the%20health%20sciences&f=false.

[24] Pourhoseingholi MA, Vahedi M, Rahimzadeh M (2013). Sample size calculation in medical studies. Gastroenterol Hepatol Bed Bench.

[25] (2023). WHO. WHOQOL: Measuring Quality of Life, Arabic_WHOQOL-BREF. https://www.who.int/tools/whoqol/whoqol-bref/docs/default-source/publishing-policies/whoqol-bref/arabic-whoqol-bref.

[26] Skevington SM, Tucker C (1999). Designing response scales for cross-cultural use in health care: data from the development of the UK WHOQOL. Br J Med Psychol.

[27] (2017). Underactive Thyroid: Deciding Whether Or Not to Treat Subclinical Hypothyroidism. https://www.ncbi.nlm.nih.gov/books/NBK279600/.

[28] Hueston WJ (2001). Treatment of hypothyroidism. Am Fam Physician.

[29] Medici BB, Lerche la Cour J, Knop FK (2021). Predictors of improvement in quality of life when treating hypothyroidism. J Thyroid Res.

[30] Feller M, Snel M, Moutzouri E (2018). Association of thyroid hormone therapy with quality of life and thyroid-related symptoms in patients with subclinical hypothyroidism: a systematic review and meta-analysis. JAMA.

[31] Högqvist Tabor V, Högqvist Tabor M, Keestra S, Parrot JE, Alvergne A (2021). Improving the quality of life of patients with an underactive thyroid through mHealth: a patient-centered approach. Womens Health Rep (New Rochelle).

[32] Roberts N (2023). Roberts N: British Thyroid Foundation Newsletter: psychological problems in thyroid disease. British Thyroid Foundation Newsletter.

[33] Saravanan P, Chau WF, Roberts N, Vedhara K, Greenwood R, Dayan CM (2002). Psychological well-being in patients on 'adequate' doses of l-thyroxine: results of a large, controlled community-based questionnaire study. Clin Endocrinol (Oxf).

[34] Ha NT, Duy HT, Le NH, Khanal V, Moorin R (2014). Quality of life among people living with hypertension in a rural Vietnam community. BMC Public Health.

[35] Rakhshan M PhD, Ghanbari A BS, Rahimi A MS, Mostafavi I BS (2017). A comparison between the quality of life and mental health of patients with hypothyroidism and normal people referred to Motahari Clinic of Shiraz University of Medical Sciences. Int J Community Based Nurs Midwifery.

[36] Shivaprasad C, Rakesh B, Anish K, Annie P, Amit G, Dwarakanath CS (2018). Impairment of health-related quality of life among Indian patients with hypothyroidism. Indian J Endocrinol Metab.

[37] Martino G, Caputo A, Vicario CM (2021). Interrelations between mental health, generic and thyroid-related quality of life in patients with Hashimoto’s thyroiditis receiving levothyroxine replacement. Mediterr J Clin Psychol.

[38] Al-Surimi K, Al-Harbi I, El-Metwally A, Badri M (2019). Quality of life among home healthcare patients in Saudi Arabia: household-based survey. Health Qual Life Outcomes.

[39] Bathla M, Singh M, Relan P (2016). Prevalence of anxiety and depressive symptoms among patients with hypothyroidism. Indian J Endocrinol Metab.

[40] Han M, Choi S, Kim S, Ko A, Son JS, Park SM (2020). Association of thyroid status with health-related quality of life in Korean older adults. Korean J Fam Med.

